# Correction: Izubuchi et al. Antitumor Effects of PD-1 Blockade Combined with Mild Hyperthermia in a Murine Osteosarcoma Model. *Biomedicines* 2026, *14*, 341

**DOI:** 10.3390/biomedicines14071520

**Published:** 2026-07-07

**Authors:** Yuya Izubuchi, Naoi Hosoe, Takaaki Tanaka, Yumiko Watanabe, Tatsunobu Kobayashi, Hideaki Nakajima, Hiroyasu Kidoya, Akihiko Matsumine

**Affiliations:** 1 Department of Orthopaedics and Rehabilitation Medicine, Faculty of Medical Sciences, University of Fukui, 23-3 Matsuoka Shimoaizuki, Eiheiji-Cho, Yoshida-Gun, Fukui 910-1193, Japan; izubuchi@u-fukui.ac.jp (Y.I.); tanaka-t@u-fukui.ac.jp (T.T.); watayu@u-fukui.ac.jp (Y.W.); t5884@u-fukui.ac.jp (T.K.); nhideaki@u-fukui.ac.jp (H.N.); 2 Department of Integrative Vascular Biology, Faculty of Medical Science, University of Fukui, 23-3 Matsuoka-Shimoaizuki, Eiheiji-Cho, Yoshida-Gun, Fukui 910-1193, Japan; hosoe@g.u-fukui.ac.jp (N.H.); kidoya@u-fukui.ac.jp (H.K.)

**Text Correction**

In the original publication [1], in Section 4 “Discussion”, the following sentence “In the present study, intratumoral temperature was not directly measured, but previous hyperthermia experiments using the same heating protocol as in the present study have been conducted in various tumor models [42,43]” was considered incorrect and misleading, as the heating protocols utilized in this study were not identical in terms of set up and configurations to those adopted in the cited papers. To improve transparency and help readers better understand the methodological context of the study, while maintaining the integrity of the scientific conclusions, a major correction has been issued.

In paragraph 2.6 “Hyperthermic Treatment for Mice”, the sentence “In addition, the capsule openings were sealed to prevent water ingress, and no external airflow was applied, which differs from previously reported setups” was added to highlight key procedural differences.

In Section 4 “Discussion”, one sentence was revised and two new sentences were added. The sentence “In the present study, intratumoral temperature was not directly measured, but previous hyperthermia experiments using the same heating protocol as in the present study have been conducted in various tumor models [42,43]” was revised with more accurate wording as “In the present study, intratumoral temperature was not directly measured, but previous hyperthermia experiments using a similar experimental concept of localized hyperthermia have been conducted in various tumor models [42,43]”. Also, the following sentences were added: “Unlike previous reports, our protocol involved sealing the capsule openings to prevent water ingress and did not incorporate external airflow for systemic temperature regulation. These differences may influence temperature distribution and represent methodological variations from the original reports” to highlight key procedural differences, and “Furthermore, because intratumoral temperature was not directly measured and the heating setup differed in certain technical aspects from previous reports, the interpretation of thermal effects should be made with appropriate caution” to emphasize the limitation regarding the absence of direct intratumoral temperature measurement.

The authors state that the scientific conclusions are unaffected. This correction was approved by the Academic Editor. The original publication has also been updated.
